# Hypofractionated Volumetric-Modulated Arc Radiotherapy for Patients With Non-Small-Cell Lung Cancer Not Suitable for Surgery or Conventional Chemoradiotherapy or SBRT

**DOI:** 10.3389/fonc.2021.644852

**Published:** 2021-06-16

**Authors:** Junyue Shen, Dan Yang, Mailin Chen, Leilei Jiang, Xin Dong, Dongming Li, Rong Yu, Huiming Yu, Anhui Shi

**Affiliations:** ^1^ Department of Radiation Oncology, Key Laboratory of Carcinogenesis and Translational Research (Ministry of Education/Beijing), Peking University Cancer Hospital & Institute, Beijing, China; ^2^ Departments of Radiology, Key Laboratory of Carcinogenesis and Translational Research, Ministry of Education, Peking University Cancer Hospital and Institute, Beijing, China

**Keywords:** radiation therapy, hypofractionation, NSCLC, tumor control, toxicities

## Abstract

**Background:**

Hypofractionated radiotherapy (HypoRT) has been used to pursue an alternative treatment regimen for patients with non-small-cell lung cancer (NSCLC) who are not eligible for stereotactic ablative radiotherapy (SABR), surgery or concurrent chemoradiotherapy (CCRT) and has shown good local control and safety. We analyzed the feasibility of using volumetric-modulated arc radiotherapy (VMAT) with the simultaneous integrated boost (SIB) technique to achieve high local control with few treatment-related toxicities.

**Patients and Methods:**

A total of 55 patients with stage I-IV NSCLC who were not candidates for SABR, surgery or CCRT were included in the present study. All patients received a prescribed dose of 60 to 66 Gy in 15 fractions. Local progression-free survival (LPFS), PFS, overall survival (OS), and toxicities were retrospectively analyzed.

**Results:**

Thirty-three patients (60.0%) had stage IV or recurrent disease in this study. The median follow-up time was 8 months (interquartile range: 5.0-16.3 months). The 1-year and 2-year OS rates were 84.3% and 69.9%, and the 1-year and 2-year LPFS rates were 91.0% and 63.0%. The median OS (mOS) and median LPFS (mLPFS) were not reached, and median PFS (mPFS) was 15 months. Twenty-eight (51.9%) patients had disease progression at the time of analysis. Of these, 7 (13.0%), 7 (13.0%) and 21 (38.9%) had local recurrence, locoregional failure and distant metastasis, respectively. All cases of local recurrence were found within the SIB region. Four patients had grade 2-3 pneumonitis, and 8 patients had grade 2-3 esophagitis. Patients with grade 2-3 esophagitis had significantly higher maximum dose and dose to 5 cm^3^ volume to esophagus than those with grade 0-1 esophagitis. No grade 4 or higher toxicity was observed.

**Conclusion:**

The 60 to 66 Gy in 15 fractions RT regimen provides favorable local control and survival with well-tolerated toxicities. Hypofractionated VMAT+SIB is an alternative treatment option for patients with NSCLC who cannot tolerate standard definitive therapy.

## Introduction

Radiation therapy has been commonly used in the treatment of patients with non-small-cell lung cancer (NSCLC). Stereotactic body radiation treatment (SBRT), known as stereotactic ablative radiotherapy (SABR), is being increasingly accepted as a definitive treatment strategy for patients who are not candidates for surgery or refuse surgical resection. Concurrent chemoradiotherapy (CCRT) is the standard treatment option for inoperable, locally advanced NSCLC.

However, some patients with early-stage, inoperable NSCLC are not candidates for SBRT due to the size or location of the lesion (1). In addition, for patients with a poor performance status (PS), CCRT is not always tolerable because the risk of adverse effects outweighs potential treatment benefits. For such patients, RT alone with standard fractionation (60-63 Gy in 30-33 fractions) has been used as front-line therapy, although with a poorer overall survival (OS) and local control. In recent years, increasing attention has been focused on hypofractionated radiotherapy (HypoRT), taking into account its shorter treatment time. With regard to early-stage NSCLC, HypoRT with a treatment dose of 60 Gy in 3-Gy fractions and 48-60 Gy in 4-Gy fractions has indicated potent 2-year local control (2–5). However, for patients with locally advanced NSCLC, or with a poor PS and metastasis, the 45 Gy in 3-Gy fractions HypoRT regimen has only been indicated to provide comparable local control to the standard RT regimen (6, 7). Thus, alternative radiation regimens were explored. More recently, a phase I dose-escalation trial demonstrated that doses up to 60 Gy in 4-Gy fractions were well tolerated in NSCLC patients with a poor PS (8). A previous study reported a favorable outcome with the 60 Gy in 4-Gy fractions regimen compared to the 60-66 Gy (9) in 3-Gy fractions regimen (10, 11). Although HypoRT at a dose of 60 Gy in 4-Gy fractions still showed no difference in OS or progression-free survival (PFS) compared to conventional RT (60 Gy/30 fractions) in a recent interim analysis of a phase III trial, it consumed half of the time and caused less toxicity (12).

HypoRT has been used at our institution for patients with stage I-III NSCLC who are not candidates for surgery, CCRT or SBRT, either due to a comorbidity or tumor size and location. In addition, stage IV patients with a low burden of metastases and a good PS are eligible for this strategy. We used the simultaneous integrated boost (SIB) technique to achieve a gross tumor volume (GTV) dose of over 60 Gy and a planning target volume (PTV) dose of over 45 Gy in 15 fractions. We report our experience with hypofractionated volumetric-modulated arc radiotherapy (VMAT) + SIB, including local control and toxicities.

## Patients and Methods

### Patients Characteristics

This retrospective study was approved by the institutional review board. A total of 55 patients who visited our hospital between December 2017 and November 2020 were included in this retrospective study. The inclusion criteria were as follows: 1) patients with pathologically confirmed NSCLC; 2) patients with stage I-III NSCLC who were not candidates for surgery, SBRT or CCRT; and 3) patients with stage IV NSCLC who had a low burden of metastases and a good PS.

### Target Volume and Organ at Risk Delineation

All patients were immobilized in the supine position, and contrast enhanced computed tomography (CT) scans were performed with 3 mm thick slices. The GTV was contoured unless the internal gross target volume (IGTV) was contoured to encompass the tumor throughout the respiratory cycle if a four-dimensional simulation was undertaken. The clinical target volume (CTV), including the primary tumor and metastatic lymph nodes, with a margin of 0.6 to 0.8 cm for microscopic extension of the primary tumor and 0.5 cm for regional lymph nodes (with adaption to the anatomy), was expanded with an additional 5-mm margin to create the PTV. OARs, such as the trachea, great vessels, spinal cord, esophagus, heart and lung, were outlined on each image.

### Planning Techniques and Objectives

In all patients, radiation therapy was delivered using the VMAT+SIB technique. The GTV/IGTV was prescribed at a dose of 60-66 Gy, and the PTV was prescribed at a dose of 45-60 Gy. All the treatment plans were designed to deliver prescription doses in 15 fractions using the Eclipse treatment planning system with a 6-MV photon beam from a Varian linear accelerator (True Beam or Edge). All plans aimed to achieve a minimum dose larger than 95%. Given the lack of established dose constraints for OARs using this regimen, we defined the primary objectives as follows based on our experience: trachea: Dmax ≤ 54 Gy; heart: Dmax ≤ 54 Gy; great vessels: Dmax ≤ 60 Gy; esophagus: Dmax ≤ 54 Gy; spinal cord: Dmax ≤ 37.5 Gy; ribs: Dmax ≤ 60 Gy; lungs: V20 ≤ 30%; and mean lung dose ≤ 15 Gy.

### Follow-Up Protocol

Patients were examined once per week during the RT course. Patients routinely underwent chest and abdominal contrast-enhanced CT, cranial magnetic resonance imaging (MRI) and superficial lymph node ultrasound 4-6 weeks after the RT course, every 3 months thereafter for the first 2 years, and every 6 months for the next 3 years. During the follow-up, treatment-related toxicities were evaluated with the Common Terminology Criteria for Adverse Events, version 5.0.

Locoregional failure was defined as a recurrent or progressed lesion within the ipsilateral lung, hilum or mediastinum after RT, while local failure was determined if occurring within the PTV. Failure was further defined as within-SIB-field after a side-by-side comparison of the diagnostic image with the radiation treatment plan if the center of the failure was encompassed by the SIB field. Patients were advised to undergo positron emission tomography (PET) and tissue biopsy if locoregional recurrence was suspected; however, cross-sectional imaging alone was also eligible to determine failure in some patients.

### Statistical Analysis

All statistical analyses were performed using IBM SPSS Statistics software, version 23.0.0 (Chicago, IL). OS, PFS, and local PFS (LPFS) were evaluated using the Kaplan-Meier method, and the log-rank test was used to assess the equality of the survivor function across groups. The time-to-event was defined from the start of the RT course to the occurrence of the event. The dosimetric differences between groups were analyzed using student T-test. Differences were considered statistically significant at P ≤ 0.05.

## Results

### Patient, Tumor and Treatment Characteristics

Detailed patient, tumor and treatment characteristics are presented in **Table 1**. A total of 55 patients were included in this study, with one patient experiencing myocardial infarction during the RT course. Given his past medical history of coronary heart disease, the myocardial infarction was not considered a treatment-related toxicity, and such patient was not included in the toxicity and survival analyses. Most of the patients had metastatic or recurrent disease (60.0%). Patients with early-stage NSCLC were either inoperable or refused surgical resection and were not candidates for SABR. Patients with locally advanced NSCLC were inoperable and could not tolerate CCRT. Twenty (36.4%) patients had central disease. A central lesion was defined according to International Association for the Study of Lung Cancer (IASLC) guidelines as a GTV located within 2 cm of the bronchial tree, great vessels, heart or spinal cord. All patients completed the RT course and received the prescribed dose. All patients received the prescribed dose, with 8 (14.5%), 21 (38.2%), 1 (1.8%), 5 (9.1%), 20 (36.4%) patients receiving the 60 Gy/45 Gy/15 fractions, 60 Gy/54 Gy/15 fractions, 66 Gy/45 Gy/15 fractions, 66 Gy/54 Gy/15 fractions, and 66 Gy/60 Gy/15 fractions RT regimens, respectively. The majority of the patients (42, 76.4%) received chemo-agent therapy before HypoRT; however, only 16 patients had an interval to the start of RT from the end of the last cycle of chemo-agent therapy of less than 1 month, and 8 of whom received targeted therapy. Thirteen patients received concurrent targeted therapy during the RT course, of theses, 10, 1, 1, and 1 of whom received EGFR-tyrosine kinase inhibitors (TKIs), anlotinib, crizotinib and alectinib, respectively. Post-RT chemo-agent therapy was undertaken in 30 (54.5%) patients, with 12 initiating treatment for disease progression. No patients received immunotherapy before or during the RT course. Eight patients received immunotherapy after RT, with 5 initiating treatment for disease progression and 3 for maintenance therapy.

**Table 1:** Patient, dosimetry and treatment characteristics.

Characteristic	Patients (n = 55)
Age (yr)	
Median	65.7
Range	32-88
Gender	
Female	24 (43.6)
Male	31 (56.4)
Smoking history (pack-year)	
<30	32 (58.2)
≥30	23 (41.8)
ECOG PS	
0-1	46 (83.6)
2-4	9 (16.4)
Histologic type	
Squamous cell carcinoma	18 (32.7)
Adenocarcinoma	24 (43.6)
Others	13 (23.7)
Gene mutation	
EGFR	14 (25.5)
ALK	2 (3.6)
ROS	1 (1.8)
None detected	11 (20.0)
Unknown	27 (29.1)
PD-L1 expression	
<1%	5 (9.1)
1%-49%	4 (7.3)
≥50%	2 (3.6)
Unknown	44 (80.0%)
AJCC 8^th^ stage	
I	4 (7.3)
II	5 (9.1)
III	13 (23.6)
IV	29 (52.7)
Recurrent	4 (7.3)
Location	
Central	20 (36.4)
Peripheral	35 (63.6)
Target Volume	
Primary	34 (61.8)
Primary and Lymph nodes	21 (38.2)
PTV volume (cm^3^)	
Median	149.4
Range	20.3 - 988.4
GTV/IGTV volume (cm^3^)	
Median	45.6
Range	2.3 – 438.2
RT regimen (GTV/PTV/fractions)	
60 Gy/54 Gy/15 fractions	21(38.2)
66 Gy/60 Gy/15 fractions	20 (36.4)
Others	14 (25.4)
Chemo-agent therapy^a^	
Induction	16 (29.1)
Concurrent	13 (23.6)
Post-RT	30 (54.5)

Data presented as n (%) or mean ± standard deviation.

^a^including chemotherapy, targeted therapy and immunotherapy.

AJCC, American Joint Committee on Cancer; ECOG PS, Eastern Cooperative Oncology Group performance status; PTV, planning tumor volume; GTV, gross tumor volume; IGTV, interval gross tumor volume; RT, radiotherapy.

### Survival and Patterns of Failure

**Figure 1:**
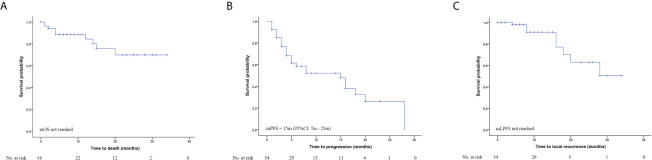
Overall survival **(A)**, Progression free survival **(B)** and Local progression free survival **(C)** for all patients.

A total of 54 patients were included in the survival analysis. The median follow-up time from the start of RT was 8 months (interquartile range: 5.0-16.3 months). The mOS was not reached, and the 1-year and 2-year OS rates were 84.3% and 69.9% (**Figure 1A**). Cancer progression was the most common cause of death in this study. The cause of death was pulmonary embolism in one patient and disseminated intravascular coagulation in another patient.

The mPFS was 15 months, with 12-month, 18-month and 24-month PFS rates of 52.1%, 32.8% and 26.2%, respectively (**Figure 1B**). The median LPFS was not reached, with 1-year and 2-year LPFS rates of 91.0% and 63.0% (**Figure 1C**). **Figure 1** shows the Kaplan-Meier curves for OS, PFS and LPFS for all patients.

**Figure 2:**
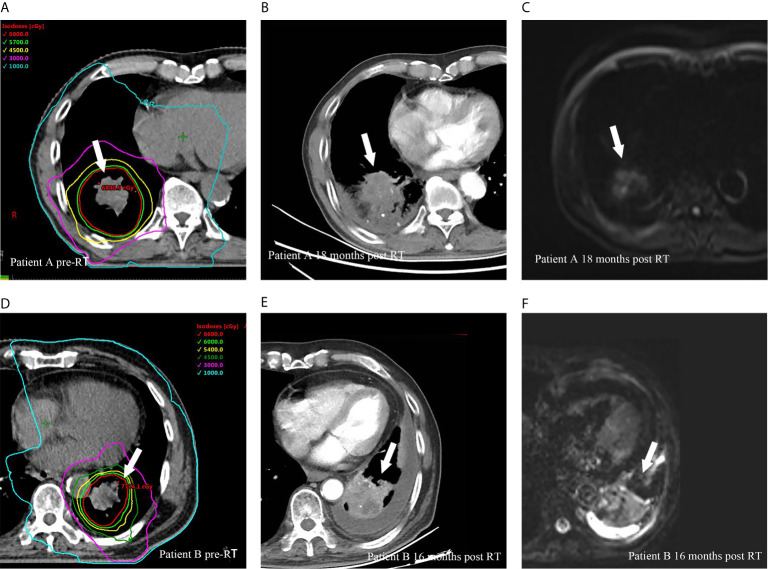
Representative cases of local recurrences within SIB region for two patients after HypoRT treatment. Planning computed tomography fused with isodose levels before RT courses for patient A **(A)**, diagnostic computed tomography **(B)** and magnetic resonance diffusion weighted imaging **(C)** showed a local recurrence (arrows) 18 months after RT. **(D–F)** presented the planning imaging, the local recurrence in diagnostic computed tomography and magnetic resonance diffusion weighted imaging 16 months after RT for patient B.

Twenty-eight (51.9%) patients had disease progression at the time of analysis. Of these, 7 (13.0%), 7 (13.0%) and 21 (38.9%) had local recurrence, locoregional failure and distant metastasis, respectively. All local recurrences were found within the SIB region, and representative examples are illustrated in **Figure 2**.

The dose to the GTV/IGTV and PTV had no significant influences on OS, PFS and LPFS. No significant differences in survival or failure patterns were found among the RT regimens.

### Toxicity

The treatment-related toxicities observed in this study are presented in **Table 2**. Three patients had grade 2 pneumonitis. One patient had fever and persistent cough along with radiologic changes on the thoracic CT imaging 60 days after the end of RT course, and the other two had shortness of breath and cough 127 and 149 days after RT and then found radiologic changes on CT imaging. All patients received glucocorticoid therapy and recovered within 2 months. One patient had grade 3 pneumonitis 76 days after RT and recovered within 3 months after hospitalization. Five patients had grade 2 esophagitis, and 3 patient had grade 3 esophagitis. No grade 4 or higher toxicity was observed. No significant dosimetric difference was found between patients with grade 0-1 pneumonitis and grade 2-3 pneumonitis. Patients experiencing grade 2 and 3 esophagitis had significantly higher maximum dose (47.43 Gy ± 17.03 Gy vs. 30.46 Gy ± 15.69 Gy, p = 0.007) and D5cc (30.76 Gy ± 15.13 Gy vs. 15.76 Gy ± 11.04 Gy, p = 0.003) to esophagus than those experiencing grade 0 and 1 esophagitis. The mean esophageal dose in patients with grade 2 and 3 esophagitis appeared to be greater than those who had grade 0 and grade 1 esophagitis (p = 0.079) (**Table 3**). The sizes of the PTV and GTV/IGTV were not related to pneumonitis or esophagitis. Late treatment-related toxicities were not analyzed in all patients given the short follow-up time. Three and 1 patients observed grade 1 and 2 dysphagia, and 6 patients had grade 1 pulmonary fibrosis.

**Table 2:** Toxicity.

Toxicities	CTCAE, version 5.0				2-3
	0	1	2	3	
Acute toxicities (n=54)					
fatigue	32 (59.3)	14 (25.9)	7 (13.0)	1 (1.9)	8 (14.8)
cough	34 (63.0)	17 (31.5)	3 (5.6)	0	1 (1.9)
pneumonitis	37 (68.5%)	13 (24.1)	3 (5.6)	1 (1.9)	4 (7.4)
esophagitis	44 (81.5)	2 (3.7)	5 (9.3)	3 (5.6)	8 (14.8)
nausea and emesis	38 (70.4)	7 (13.0)	8 (14.8)	1 (1.9)	9 (16.7)
Late toxicities (n=50)					
dysphagia	46 (92.0%)	3 (6.0%)	1 (2.0%)	0	1 (2.0%)
pulmonary fibrosis	42 (84.0%)	8 (16.0%)	0	0	0

Data presented as n (%).

CTCAE, Common Terminology Criteria for Adverse Events.

**Table 3:** Dose statistics stratified by toxicities grade.

	Grade 2-3	Grade 0-1	P value
Pneumonitis	n = 4	n = 51	
Mean lung dose	7.44 ± 1.96	8.04 ± 3.65	0.748
V5	32.9 ± 13.8	32.2 ± 14.6	0.926
V15	18.8 ± 8.0	16.8 ± 9.0	0.662
V18	13.6 ± 4.2	14.5 ± 7.9	0.809
V20	12.2 ± 4.1	13.2 ± 7.2	0.786
Esophagitis	n = 8	n = 46	
Mean esophagus dose	15.05 ± 9.59	8.02 ± 5.46	0.079
Maximum dose	47.43 ± 17.03	30.46 ± 15.69	0.007
D5cc	30.76 ± 15.13	15.76 ± 11.04	0.003

Data presented as average ± standard deviation. V5, volume of target receiving at least 5 Gy given as a percent of total lung; V15, volume of target receiving at least 15 Gy given as a percent of total lung; V18, volume of target receiving at least 18 Gy given as a percent of total lung; V20, volume of target receiving at least 20 Gy given as a percent of total lung; D5cc, dose to 5 cm^3^ volume.

## Discussion

HypoRT has been investigated in an increasing number of studies in recent years due to its short treatment time. The potential advantages or the shorter treatment time may be threefold: 1) the short RT time may minimize the negative influence of rapid tumor cell proliferation (6); 2) the shorter treatment time can be easier to tolerate for patients with a poor PS; and 3) hypofractionated regimens may be particularly suitable for patients with weak financial repayment ability given their lower treatment costs (although the third advantage will only be considered if HypoRT has acceptable outcomes and treatment-related toxicities).

HypoRT was initially used for patients with NSCLC with a poor PS (13). Nguyen et al. (6) compared a hypofractionated regimen of 45 Gy in 15 fractions to the standard RT regimen of 60-66 Gy over 6 weeks in patients with stage II-III NSCLC. They demonstrated that HypoRT had comparable OS and locoregional control to standard RT despite the significantly poor PS found in HypoRT patients. Moreover, no significant differences in either acute or late toxicity with regard to pulmonary and esophageal toxicities were found between the two groups. A phase I dose-escalation trial (8) indicated that HypoRT consisting of 60 Gy in 15 fractions is generally well tolerated in patients with stage II to IV NSCLC and a poor PS. Furthermore, an interim analysis of a phase III randomized study evaluating survival outcomes in a comparison of standard RT versus HypoRT indicated that the 60 Gy in 15 fractions RT regimen had equivalent OS and PFS outcomes to the conventional RT regimen in patients with stage II-III NSCLC and a poor PS. They also found that fewer grade 3-5 toxicities were observed in the HypoRT arm (12).

More recently, the use of the HypoRT regimen has not been limited to patients with a poor PS (14, 15). Pollom et al. (9) explored HypoRT in patients with stage II-IV NSCLC who were not eligible for surgery, CCRT or SBRT. Most of the patients received 60 Gy in 15 fractions in this study. The mOS was 15.1 months, with a 1-year OS rate of 63% and a 1-year PFS rate of 22.5%. The cumulative incidence of in-field failure at 12 months was 16.1%. Their local control results compared favorably to the outcomes with 60-66 Gy in 3-Gy fractions (10, 11). Swanick et al. (16, 17) investigated HypoRT using the IMRT+SIB technique in a similar group of patients to those examined in the Pollom study. All patients received IGTV doses of 52.5 to 60 Gy and PTV doses of 45 to 52.5 Gy, and most patients received a RT regimen consisting of 52.5 Gy to the IGTV and 45 Gy to the PTV. The mOS was 9.0 months, with 3-, 6-, and 12-month OS rates of 86%, 66%, and 34%, respectively, and 3-month, 6-month, and 12-month LPFS rates of 92%, 78%, and 60%, respectively. Furthermore, they found that 17 (24%) patients had local failure, and all but 1 failure occurred within the high-dose region. These results led to the more frequent use of HypoRT in patients who were not candidates for surgery, CCRT or SBRT in our institutions. The VMAT+SIB technique has been used in our institution in recent years for patients with NSCLC. Since both the 45 Gy in 15 fractions and 60 Gy in 15 fractions RT regimens showed comparable OS, local control, and toxicities to the standard RT regimen and local recurrence was found mostly in the SIB region, we have been using the VMAT+SIB technique to prescribe a high dose to the GTV and a relatively low dose to the PTV to provide satisfactory local control and few toxicities.

All patients received prescribed doses of 60-66 Gy to the IGTV/GTV and 45-60 Gy to the PTV in this study, and the majority of patients in our study received either 66 Gy to the IGTV/GTV and 60 Gy to the PTV (n = 20, 36.4%) or 60 Gy to the IGTV/GTV and 54 Gy to the PTV (n = 21, 38.2%). The 1-year OS rate (84.3% vs. 63%), PFS rate (52.1% vs. 22.5%) and LPFS rate (91% vs. 83.9%) were better than those in Pollom group study. Although more patients in our study had stage III and stage IV disease, our patients had younger age, better performance status and smaller PTV volume. In addition, 10 patients received concurrent EGFR-TKIs in our study. Given the recent published results in RECEL study (18), concurrent EGFR-TKIs may result in better prognosis. We obtained a higher LPFS rate at 12 months than Swanick study (91% vs. 60%). Although we prescribed a higher dose to the GTV and PTV than did Swanick (most patients received 52.5 Gy to the IGTV and 45 Gy to the PTV), we hypothesize that this difference is likely attributed to the much smaller size of the PTV observed in our study (149.4 cm3 vs. 421.2 cm3). These findings indicated that better performance status and reasonable tumor burden could be important selection criteria when using this RT regimen. Moreover, we found no significant difference on OS, PFS and LPFS between patients received dose over 60 Gy to GTV/IGTV and those received dose ≤ 60 Gy to GTV/IGTV. Our finding is in line with previous studies where have reported inconsistent results between improved tumor control and a prescribed dose over 60 Gy to the tumor volume under conventional RT regimens (19, 20). Despite the lack of prospective dose-escalation studies investigating HypoRT, our results implicate that tumor control is not improved by irradiating tumors at doses over 60 Gy in a 4-Gy per fraction regimen; however, further studies need to be performed to answer this question.

We found only 1 case of grade 3 pneumonitis and 3 cases of grade 3 esophagitis and no cases of grade 4 or 5 toxicities in this study. In accordance with previous studies (8, 9, 21), we found that patients with grade 2-3 esophagitis had significantly higher maximum dose and D5ccto esophagus than those experiencing grade 0-1 esophagitis. However, we found no significant dosimetric differences in pulmonary profiles, e.g., V18 of the lungs. Late toxicities in the lungs and esophagus were assessed in 50 patients who were followed up for more than 3 months, only 3 and 1 patients observed grade 1 and 2 dysphagia, and 6 patients had grade 1 pulmonary fibrosis. Of note, 10 (20%) of these patients were followed up for only 4 or 5 months.

Several limitations to the present study should be acknowledged. First, this was a retrospective study with a relatively short follow-up time. Although short-term outcomes and acute toxicities were assessed, long-term survival and late toxicities could be more interesting given the radiobiological nature of HypoRT. Second, the present study was conducted on a small sample size with multiple confounding factors that may have influenced the outcomes, including clinical stage, tumor volume and chemotherapy options. Third, local failure was determined primarily from CT imaging. MRI, histologic confirmation and PET-CT were not always undertaken except when considered necessary.

In summary, the 60 to 66 Gy in 15 fractions RT regimen provides favorable local control and survival with well-tolerated toxicities. Hypofractionated VMAT+SIB is an alternative treatment option for patients with NSCLC who cannot tolerate SABR, surgery or CCRT.

## Data Availability Statement

The raw data supporting the conclusions of this article will be made available by the authors, without undue reservation.

## Ethics Statement

The studies involving human participants were reviewed and approved by Key Laboratory of Carcinogenesis and Translational Research (Ministry of Education/Beijing), Peking University Cancer Hospital & Institute. The patients/participants provided their written informed consent to participate in this study. Written informed consent was obtained from the individual(s) for the publication of any potentially identifiable images or data included in this article.

## Author Contributions

JS, DY, and MC finished the majority of data entry and data analysis, and composed the manuscript. These three authors have contributed equally to this work and share first authorship. LJ and XD reviewed the database prior to the data analysis. DL, RY, and HY provided part of the cases and profession advises of the treatment of NSCLC. AS provided most of the cases and gave advises to data analysis and outcomes interpretation. All authors contributed to the article and approved the submitted version.

## Funding

This study was funded by the Beijing Health and Health Science Technology Achievements and Appropriate Technology Promotion Project (No. BHTPP202026).

This study was funded by Chinese Society of Clinical Oncology (CSCO) – Linghang cancer research (No. Y-2019AZMS-0519).

## Conflict of Interest

The authors declare that the research was conducted in the absence of any commercial or financial relationships that could be construed as a potential conflict of interest.
